# 

**Published:** 1916-05-20

**Authors:** 


### PASSING EVENTS AND TOPICS.

### Medical Service of the American Army.

In connection with the reorganisation of the United States Army it has been resolved to provide for seven medical officers per thousand of line troops.

### Support for a M.O.H.

In consequence of a public attack upon Dr. Maxwell Ross, the county medical officer of Dumfries, the County Council and its Public Health Committee have passed a resolution expressing their continued confidence in Dr. Ross in his administration of the Public Health Acts.

### French School Buildings as Hospitals.

At the beginning of the present year the French Military Medical Service occupied 386 public school buildings and 244 private school buildings, the majority of which had been transformed into auxiliary hospitals. Most of these are now being restored to their original use.

### Shell Explodes in Hospital.

A visitor at Linlithgow Hospital was injured severely by the explosion of a shell handed to him by a wounded soldier.

### The Mental After-Care Association.

The annual report claims that the charity is doing work untouched by any other body in the field of philanthropic enterprise, that the labours of the executive on behalf of poor persons convalescent or recovered from institutions for the insane is often exceptionally difficult, and that many patients would have no possibility of relief from their perplexity or trouble but for the help of the Association. The charity was founded by a well-known chaplain of Colney Hatch in 1879, and last year dealt with 379 applicants, expending £1,251. The accounts published are rather unintelligible, a sort of hybrid between a balance-sheet and an income and expenditure account.

### HOSPITAL RESULTS IN 1915.

EAST END MOTHERS' LYING-IN HOME.—The Home received last year £1,335 in training fees, but the board fear that owing to new regulations of the Central Midwives Board, materially increasing the period required of pupils training in midwifery, this source of income is threatened. Thirty "sets" (the word is the lady superintendent's) of twins were born last year, and £13 10s. was disbursed from a twin bounty fund.

KING EDWARD VII. SANATORIUM, MIDHURST.—The report refers to the twelve months from July 1914 to July 1915, during which period there was a considerable falling-off in the number of applications for admission. Seventy beds are used for soldiers suffering from pulmonary tuberculosis. Three years of continuous observation upon the use of tuberculin in diagnosis and treatment were completed at the end of the year 1914. It was then deemed advisable to discontinue this method of treatment until the immediate and subsequent results obtained at the sanatorium since the adoption of tuberculin had been more fully worked out.

ROYAL SEA-BATHING HOSPITAL, MARGATE.—No legacy was received in 1915 and no bed was endowed, but the total income, £15,990, exceeded the expenditure by £378. The arrangement with the War Office to receive wounded soldiers terminated in September last, after 153 military patients had been treated in the wards. The annual report is illustrated by a number of attractive photographs of the work.

MILLER GENERAL HOSPITAL FOR SOUTH-EAST LONDON.—The numbers of in- and out-patients have both decreased, the latter by nearly 25 per cent. The smaller number of out-patients is welcomed by the Board, and attributed to the improved financial conditions of the poorer classes, the greater extent to which they have been occupied, and the continued working of the Insurance Act, which has specially affected the maternity figures. The average cost per in-patient is practically the same as that for 1914; a decrease in the cost of drugs, for which credit is largely given to the care exercised by the medical staff, having balanced the increased cost of provisions. The total income exceeded the expenditure by £2,204, and as only £75 was received from legacies this result, in these difficult times, must be considered extremely satisfactory.

RADCLIFFE INFIRMARY, OXFORD.—The total expenditure was £1,169 more than in the previous year, and the rereceipts £2,159 more. There was, however, a deficit of £2,447 on the year's working, and as the debit balance for 1914 was £5,707 the financial position must cause anxiety to the committee of management; 453 soldiers, for whom 60 beds were provided, were admitted during the year, and the War Office grant for maintenance amounted to £2,697. For the maintenance of patients insured under the National Insurance Acts a sum of £425 was received. Twenty-three per cent. of the new out-patients belonged to approved societies. In-patients, generally, are asked to contribute something towards their cost of maintenance.

### Personalia.

Captain Henry Gilbert Peake, M.B., R.A.M.C., formerly resident surgical officer at the Salford Royal Hospital, is reported wounded.

### Medical Appointments.

Dr. Percival J. Hay has been appointed honorary ophthalmic surgeon to Sheffield Royal Hospital in the place of the late Dr. Stanley Riseley.

Dr. Alexander Campbell, M.B., Ch.B. (Edin.), D.P.H., has been appointed resident surgical officer at the Birmingham and Midland Eye Hospital. Dr. Campbell has worked under Dr. Logan Turner in the ear, nose, and throat department of the Edinburgh Royal Infirmary for the last three years.

### Annual Meetings.

Zenana Bible and Medical Mission, at King George's Hall, Central Y.M.C.A., Tuesday, May 23, 3 p.m. Chairman, The Lord Kinnaird, K.T.

Masonic Service for Hospital.—A Masonic Service in aid of the Central London Throat and Ear Hospital will be held on Sunday, May 21, at 3.30 p.m., at St. Jude's Church, Gray's Inn Road.

### Matrons' and Sisters' Appointments.

Fleet Cottage Hospital.—Miss Ruth Heatley has been appointed matron. She received her training at Bideford District Dispensary and Infirmary, North Devon, and at Manchester Royal Infirmary. She has since been sister at the Children's Hospital, Cold Ash, and at Harrow Cottage Hospital, where she later undertook holiday duties as matron. Miss Heatley has also done military nursing in France and England.

National Union of Printers and Paperworkers' Convalescent Home, Carshalton, Surrey.—Miss Louise Kingham has been appointed matron. She received her training at Lewisham Infirmary, and at the Royal Westminster Ophthalmic Hospital, and has held several sisters' appointments. She has also been assistant matron at the Royal Institution for the Blind, Edgbaston, and matron of Hayes Cottage Hospital, Middlesex.

City Hospital, Liverpool.—Miss Mabel Robinson, who received her training at Toxteth Park Poor-Law Institution, Liverpool, and later became sister there, has been appointed sister at the City Hospital, Liverpool.

Perth Royal Infirmary.—Miss Mabel Knight has been appointed theatre sister. She received her training at the Royal Hampshire County Hospital, Winchester, and at the Royal London Ophthalmic Hospital.

North Lonsdale Hospital, Barrow.—Miss Gertrude Monk has been appointed night sister. She was trained at the Royal Portsmouth Hospital, and has since been night sister at the Royal Southern Hospital, Liverpool.

St. Leonard's Hospital, Sudbury.—Miss Jessie Punshon has been appointed surgical sister. She was trained at Edmonton Union Infirmary.

**NOTE.—Particulars of Appointments for insertion in this column will be welcomed.**

Rates of Subscription: Twelve months, 6/6; Six months, 4/-; Three months, 2/-, post free to any address in the United Kingdom. Abroad, Twelve months, 8/8; Six months, 5/-; Three months, 2/6, post free.—Address The Subscription Dept., "The Hospital," The Hospital Building, 28/29 Southampton Street, Strand, London, W.C.

